# *Tityus serrulatus* Scorpion Venom-Induced Nociceptive Responses Depend on TRPV1, Immune Cells, and Pro-Inflammatory Cytokines

**DOI:** 10.3390/toxins17070332

**Published:** 2025-06-30

**Authors:** Camila R. Ferraz, Marília F. Manchope, Mariana M. Bertozzi, Telma Saraiva-Santos, Ketlem C. Andrade, Anelise Franciosi, Tiago H. Zaninelli, Julia Bagatim-Souza, Sergio M. Borghi, Denise M. Cândido, Thiago M. Cunha, Rubia Casagrande, Fábio H. Kwasniewski, Waldiceu A. Verri

**Affiliations:** 1 Department of Immunology, Parasitology and General Pathology, Londrina State University, Londrina 86057-970, Paraná, Brazilanelise.franciosi@unifil.br (A.F.); tiagozaninelli@gmail.com (T.H.Z.); fhkwas@uel.br (F.H.K.); 2 Center for Research in Health Sciences, University of Northern Paraná, Londrina 86057-970, Paraná, Brazil; 3 Arthropod Laboratory, Butantan Institute, São Paulo 05503-900, São Paulo, Brazil; 4 Center of Research in Inflammatory Diseases, Ribeirão Preto Medical School, University of São Paulo, Ribeirão Preto 14049-900, São Paulo, Brazil; 5 Department of Pharmacology, Ribeirão Preto Medical School, University of São Paulo, Ribeirão Preto 14049-900, São Paulo, Brazil; 6 Department of Pharmaceutical Sciences, Centre of Health Sciences, Londrina State University, Londrina 86057-970, Paraná, Brazil

**Keywords:** *Tityus serrulatus*, hyperalgesia, pain, neuroinflammation, TNF-α, IL-1β, NFκB

## Abstract

For centuries, researchers have been fascinated by the composition of scorpion venom and its local and systemic effects on humans. During a sting, scorpions inject peptides and proteins that can affect immune cells and neurons. While the immune and nervous systems have been studied independently in the context of scorpion stings, here we reveal part of the mechanism by which *Tityus serrulatus* venom induces hyperalgesia in mice. Through behavioral, immune, imaging assays, and mice genetics, we demonstrate evidence of neuroimmune crosstalk during scorpion stings. *Tityus serrulatus* venom induced mechanical and thermal hyperalgesia in a dose-dependent manner, as well as overt pain-like behavior. The venom directly activated dorsal root ganglia neurons and increased the recruitment of macrophages and neutrophils, releasing pro-inflammatory cytokines TNF-α and IL-1β. Blocking TRPV1^+^ neurons, TNF-α, IL-1β, and NFκB reduced the mechanical and thermal hyperalgesia, overt pain-like behavior, and the migration of macrophages and neutrophils induced by *Tityus serrulatus* venom. Collectively, *Tityus serrulatus* venom targets primary afferent nociceptive TRPV1^+^ neurons to induce hyperalgesia through the recruitment of macrophages and neutrophils and the release of pro-inflammatory cytokines.

## 1. Introduction

There are more than 2800 scorpion species worldwide. Among the 22 recognized scorpion families, Buthidae stands out as the most diverse, comprising over 80 genera, including the medically significant genus *Tityus* [1,2]. *Tityus* scorpions occur throughout parts of lower Central America, South America, and the Caribbean, with their habitat range extending from southern Costa Rica all the way to northern Argentina [3,4,5,6]. Early studies of Tityus envenoming concentrated mainly on the medically significant Brazilian species *Tityus serrulatus* (Brazilian yellow scorpion) and *Tityus bahiensis* (Brazilian brown scorpion) [7]. *Tityus serrulatus* is native to Brazil, and its venom is highly potent [8,9,10]. Considered the most lethal scorpion in South America, it accounts for the majority of deadly envenomation cases [8,11]. In addition to systemic effects induced by *Tityus serrulatus* venom, including tachycardia, diaphoresis, profuse sweating, psychomotor agitation, tremors, nausea, vomiting, sialorrhea, and either hypertension or hypotension, victims also experience intense pain at the sting site [11,12]. Initial pain management includes non-opioid analgesics and non-steroidal anti-inflammatory drugs (NSAIDs), while moderate-to-severe pain may require opioids. Adjuvant therapies such as topical lidocaine or nerve blocks can be considered. Although antivenom is administered for systemic toxicity, it does not directly alleviate pain [7,13,14]. This therapeutic gap underscores our incomplete understanding of venom’s allogenic effects and the molecular targets in nociceptive pathways.

*Tityus serrulatus* venom contains neurotoxins that act on sodium and potassium ion channels, as well as metalloproteases and antimicrobial peptides [7,9,10,11]. Envenomation by *Tityus serrulatus* triggers a systemic immune response, characterized by elevated pro-inflammatory cytokines (IL-1β, IL-6, TNF-α, IFN-γ, and IL-8) in humans, with levels correlating to clinical severity [15,16]. Animal studies demonstrate leukocytosis, neutrophil recruitment, and increased inflammatory mediators (IL-6, TNF-α, IL-10) [17]. The macrophages are key responders during *Tityus serrulatus* envenomation, releasing cytokines (IL-6, TNF-α, NO) and lipid mediators (PGE_2_, LTB_4_) via TLR/PPAR-γ pathways [18,19,20,21]. Additionally, *Tityus serrulatus* venom also activates the complement system, enhancing neutrophil chemotaxis (mediated by C5a) and exacerbating inflammation [22]. Together, cytokines, complement activation, and lipid mediator release drive severe systemic inflammation and increase the mortality risk in envenomation cases.

Despite extensive studies on the immune response, the mechanisms underlying the local pain induced by *Tityus serrulatus* venom remain poorly understood. While it is well-established that the venom modulates immune responses through cytokine production and immune cell activation, the potential involvement of neuroimmune interactions in venom-induced pain has not been thoroughly investigated.

In this study, we show that *Tityus serrulatus* venom directly activates dorsal root ganglion (DRG) neurons and promotes the recruitment of macrophages and neutrophils, alongside an increased release of pro-inflammatory cytokines such as TNF-α and IL-1β. Pharmacological and genetic approaches demonstrate that blocking TRPV1^+^ nociceptors (using TRPV1-deficient mice), as well as inhibiting NFκB, TNF-α, or IL-1β signaling, significantly reduces venom-induced mechanical and thermal hyperalgesia, spontaneous pain behavior, and immune cell infiltration. Together, our findings uncover a previously unrecognized neuroimmune axis in *Tityus serrulatus* venom-induced pain, in which TRPV1^+^ sensory neurons, immune cell recruitment, and cytokine signaling converge to drive nociceptive responses.

## 2. Results

### 2.1. Tityus serrulatus Venom Induces Dose-Dependent Mechanical and Thermal Hyperalgesia, Spontaneous Pain Behaviors, and Local Inflammatory Responses

**Figure 1:**
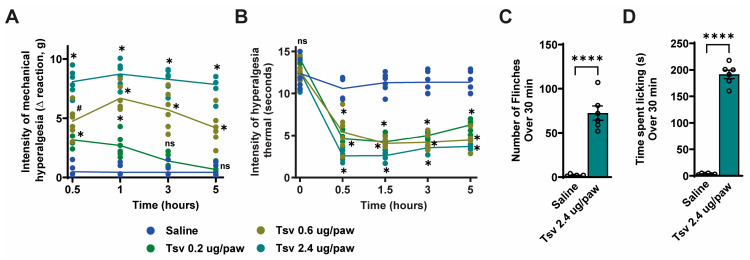
The venom of *Tityus serrulatus* triggers mechanical and thermal hyperalgesia in a dose-dependent manner in mice and induce spontaneous behaviors. Mice administered intraplantar injections of *Tityus serrulatus* venom at doses of 0.2, 0.6, or 2.4 μg in 20 μL per paw, or were given 20 μL of saline as a control. Assessments of mechanical and thermal hyperalgesia (panels (**A**,**B**)) were conducted over a 0.5–5-h period following injection. Spontaneous pain behaviors, such as paw flinching (panel (**C**)) and licking (panel (**D**)), were monitored during the first 30 min after administration. Data are shown as mean ± SEM for six mice per group, and the graphs represent findings from one of two separate experiments. Statistical significance is indicated as follows: (**A**,**B**) * *p* < 0.05 versus saline group; # *p* < 0.05 versus saline and lower venom doses (analyzed by two-way ANOVA with Tukey’s post hoc test); (**C**,**D**) **** *p* < 0.0001 versus saline (Student’s *t*-test).

To characterize the hyperalgesic effects of *Tityus serrulatus* venom, we conducted a dose-response experiment using intraplantar (i.pl.) injection of 0.2, 0.6, or 2.4 μg *Tityus serrulatus* venom. Behavioral assessments for mechanical and thermal hyperalgesia were performed at 0.5, 1, 3, and 5 h after injection (Figure 1A,B). *Tityus serrulatus* venom induced dose-dependent mechanical hyperalgesia, with the 2.4 μg dose producing the most pronounced effect across all timepoints (Figure 1A). In contrast, thermal hyperalgesia showed no significant dose-dependent differences (Figure 1B). Based on these results, we selected the 2.4 μg dose for subsequent experiments. Further evaluation revealed that this dose consistently elicited spontaneous pain behaviors (Figure 1C,D), including significant increases in both paw flinching (Figure 1C) and paw licking (Figure 1D) time compared to vehicle control.

**Figure 2:**
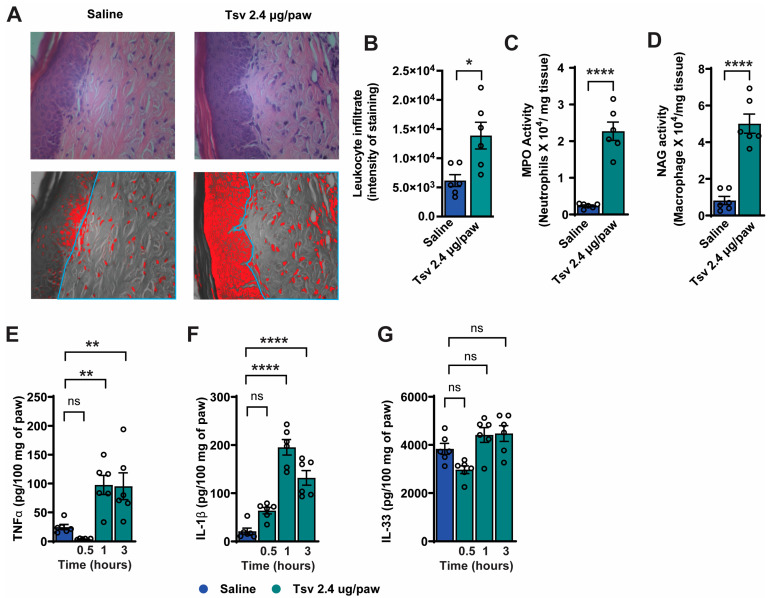
*Tityus serrulatus* venom promotes leukocyte infiltration, increases MPO and NAG enzyme activity, and promotes TNF-α and IL-1β levels in mice. Animals were given intraplantar injections of the venom (2.4 μg in 20 μL per paw) or saline (20 μL) as a control. Paw tissue was collected 5 h after injection for histopathological examination (panels (**A**,**B**)), and to assess MPO (panel (**C**)) and NAG (panel (**D**)) activity. Leukocyte accumulation was quantified as a percentage of tissue area using ImageJ 1.44 software, with highlighted regions marked in light blue. In a separate analysis (panels (**E**–**G**)), paw tissues were harvested at 0.5, 1, and 3 h after venom injection to measure the levels of pro-inflammatory cytokines TNF-α (**E**), IL-1β (**F**), and IL-33 (**G**) via ELISA. Data are expressed as mean ± SEM, with six mice per group, and represent one of two independent experiments. Statistical significance is indicated as follows: (**A**–**D**) * *p* < 0.05, **** *p* < 0.0001 compared to saline (Student’s *t*-test); (**E**–**G**) **** *p* < 0.0001, ** *p* < 0.002 versus saline (One-way ANOVA followed by Tukey’s post hoc test).

Leukocyte infiltration is indicative of inflammatory response and contributes to pain pathways [23,24]. Considering this, we examined the impact of *Tityus serrulatus* venom on this parameter (Figure 2A–D). Initially, a histopathological analysis was conducted on the skin of the hind paw skin using H&E staining (Figure 2A,B). Our findings revealed that *Tityus serrulatus venom* triggered leukocyte infiltration. To further explore whether *Tityus serrulatus venom* promotes the recruitment of neutrophils and macrophages to the paw skin, we measured MPO and NAG enzyme activities using colorimetric assays (Figure 2C,D). The results confirmed increased leukocyte infiltration, as indicated by elevated leukocyte numbers along with higher MPO and NAG activity levels. We then assessed *Tityus serrulatus* venom’s effect on the production of pro-inflammatory cytokines TNF-α, IL-1β, and IL-33 (Figure 2E,F), which are known to contribute to hyperalgesia, overt pain-like behavior, and leukocyte recruitment [25,26,27,28,29]. *Tityus serrulatus* venom significantly increased TNF-α and IL-1β levels, but not IL-33. Based on these findings, we focused subsequent experiments on determining the endogenous roles of TNF-α and IL-1β in mediating the nociceptive and inflammatory responses induced by *Tityus serrulatus* venom.

### 2.2. TNF-α, IL-1β and NFκB Inhibition Attenuates Tityus serrulatus Venom-Induced Hyperalgesia and Inflammatory Response

**Figure 3:**
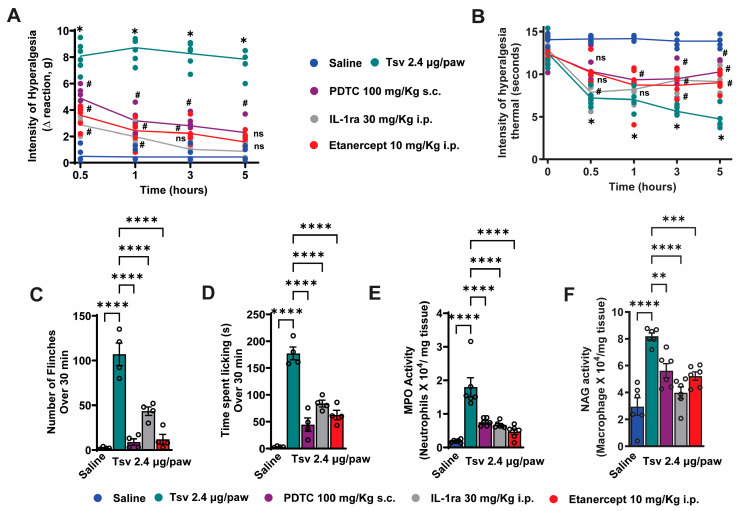
Systemic administration of NFκB, TNF-α, and IL-1β inhibitors effectively reduces the mechanical and thermal hyperalgesia, spontaneous pain behaviors, and decreased MPO and NAG enzyme activities caused by *Tityus serrulatus* venom in mice. Prior to intraplantar injection of venom (2.4 μg in 20 μL per paw), mice were pretreated with either PDTC (100 mg/kg, 100 μL subcutaneously, 30 min beforehand), IL-1 receptor antagonist (IL-1ra; 30 mg/kg, 200 μL intraperitoneally, 30 min prior), Etanercept (10 mg/kg, 200 μL intraperitoneally, administered 48 h before and again 1 h after venom), or a saline control. Assessments of mechanical and thermal hyperalgesia (panels (**A**,**B**)) were conducted between 0.5 and 5 h after injection. Spontaneous pain behaviors, such as paw flinching (**C**) and paw licking duration (**D**), were recorded during the first 30 min after venom exposure. At 5 h after injection, paw tissue was collected for analysis of MPO (**E**) and NAG (**F**) activity. Data are presented as mean ± SEM for groups of six mice for (panels (**A**,**B**,**E**,**F**)) and four mice for (panels (**C**,**D**)), with graphs showing results from one of two independent experiments. Statistical significance is as follows: (**A**,**B**) * *p* < 0.05 versus saline and treatment groups; # *p* < 0.05 versus both saline and venom-only groups (Two-way ANOVA with Tukey’s test); (**C**–**F**) **** *p* < 0.0001, *** *p* < 0.001, ** *p* < 0.002 compared to saline group (One-way ANOVA with Tukey’s post hoc analysis).

Since TNF-α and IL-1β production is regulated by NFκB activation [30,31], we investigated whether blocking NFκB, TNF-α, and IL-1β pharmacologically could reduce the inflammatory effects induced by *Tityus serrulatus* venom (Figure 3A–F). Each treatment significantly declined *Tityus serrulatus* venom-induced mechanical and thermal hyperalgesia (Figure 3A,B) diminished spontaneous pain behaviors (Figure 3C,D), and lowered MPO and NAG enzyme activities (Figure 3E,F). These findings indicate that the hyperalgesia, spontaneous pain behaviors, and leukocyte infiltration caused by *Tityus serrulatus* venom rely on the activation of NFκB and the production of TNF-α and IL-1β (Figure 3A–F).

### 2.3. Dorsal Root Ganglion (DRG) Neurons Are Activated by Tityus serrulatus Venom

**Figure 4:**
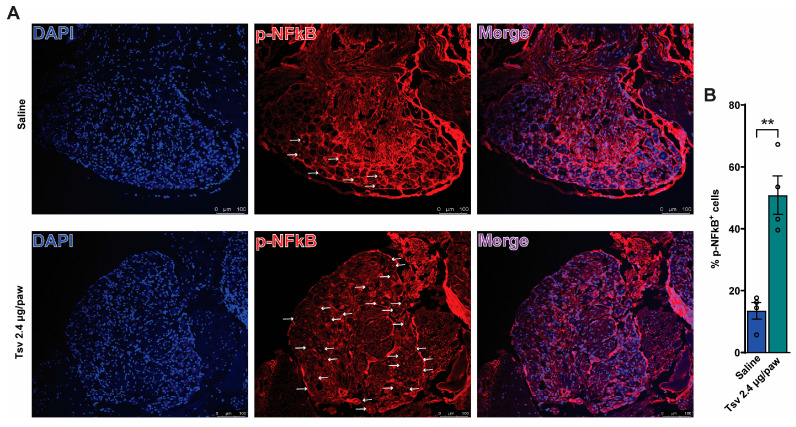
*Tityus serrulatus* venom triggers neuronal activation in mice. Animals received intraplantar injections of the venom (2.4 μg in 20 μL per paw) or a control saline solution (20 μL). *DRG neurons* (segments L4–L6) were harvested 3 h after injection for immunofluorescence analysis targeting phosphorylated NFκB (pNFκB). Representative images show pNFκB-positive cells stained in red (panel (**A**)). Quantitative evaluation included the number of pNFκB-positive cells per area and the percentage of positive cells (panel (**B**)), using images taken at 20× magnification with a 1.0 zoom factor. Data are expressed as mean ± SEM for four mice per group, and the graph displays results from one of two independent experiments. A statistically significant increase (** *p* < 0.002) was observed in comparison to the saline group (Student’s *t*-test).

To determine whether i.pl. venom injection activates nociceptive circuits, we examined pNFκB activation in DRG neurons. In the DRG neurons, pNFκB serves as an index of neuronal activation [31,32]. The analysis of colocalization was performed with pNFκB and DAPI. We observed a significant increase in the number of nuclear pNFκB+ neurons in the *Tityus serrulatus* venom group compared to the saline group (Figure 4A,B).

**Figure 5:**
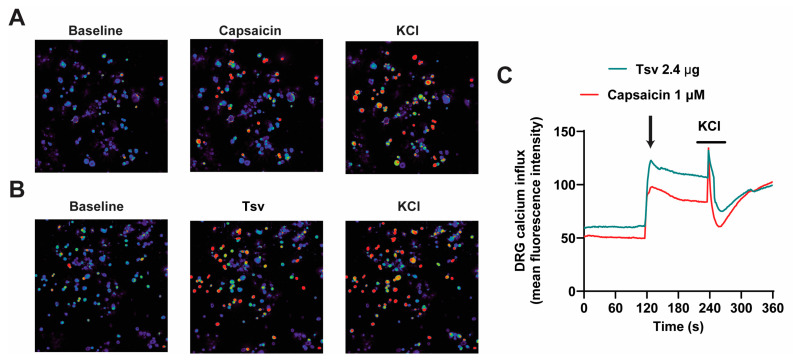
*Tityus serrulatus* venom directly stimulates sensory neurons. The figure shows representative ratiometric Fura-4 images (**A**,**B**) and corresponding calcium response traces (**C**) from DRG neurons recorded under baseline conditions and following in vitro exposure to *Tityus serrulatus* venom (2.4 μg), capsaicin (1 μM), and potassium chloride (KCl, 40 mM). Naïve DRG neurons culture was stimulated with capsaicin and after with KCl (**A**) and naïve DRG neurons culture was stimulated with *Tityus serrulatus* venom and after with KCl (**B**). Scale bars represent 50 μm. Data are presented as mean values from four DRG culture plates per group, with each plate derived from pooled neurons from 10 mice. Results are representative of two independent experiments.

Activation of nociceptive DRG neurons is typically associated with increased calcium influx because TRPV1 is permeable to calcium upon activation [32,33,34]. To determine whether DRG neurons are directly activated by *Tityus serrulatus* venom, we stimulated cultured DRG neurons with *Tityus serrulatus* venom and measured their responses using calcium imaging. Our data demonstrate that *Tityus serrulatus* venom induces calcium influx in these neurons, suggesting direct activation. Furthermore, the calcium imaging profile of *Tityus serrulatus* venom stimulation paralleled the response triggered by capsaicin, which is a TRPV1 agonist [35] (Figure 5A–C).

**Figure 6:**
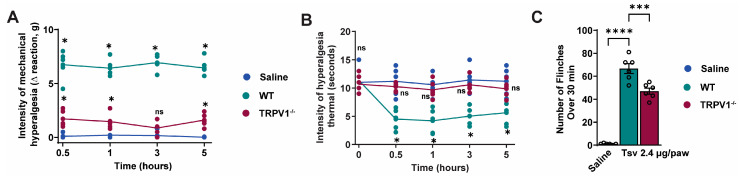
TRPV1-expressing neurons contribute to pain signaling triggered by *Tityus serrulatus* envenomation. Mice were administered intraplantar injections of *Tityus serrulatus* venom (2.4 μg in 20 μL per paw) or saline (20 μL) as a control. Mechanical (**A**) and thermal (**B**) hyperalgesia were assessed between 0.5 and 5 h after injection. Spontaneous behaviors, specifically paw flinching (**C**), were recorded within the first 30 min after venom exposure. Data are shown as mean ± SEM from six mice per group and represent one of two independent experimental replicates. Statistical analysis showed: (**A**,**B**) * *p* < 0.05 versus both saline and venom-only groups (Two-way ANOVA with Tukey’s post hoc test); (**C**) **** *p* < 0.0001 versus saline, *** *p* < 0.002 versus venom-only group (One-way ANOVA followed by Tukey’s test).

TRPV1 is an ion channel that is permeable to calcium upon activation and its expression characterizes a subpopulation of neurons that play a role in pain [35,36]. Next, we assessed the role of TRPV1+ neurons in *Tityus serrulatus* venom-induced pain (Figure 6A–C). TRPV1 deficiency significantly reduced mechanical (Figure 6A), and thermal (Figure 6B) hyperalgesia evoked by *Tityus serrulatus* venom at all time points. Moreover, TRPV1-deficient mice exhibited reduced *Tityus serrulatus* venom-induced paw flinches (Figure 6C), further confirming TRPV1’s critical role in venom-mediated pain signaling.

## 3. Discussion

Scorpion venom contains multifunctional protein and peptide toxins evolved for prey and defense [7,11,14]. Although their stings cause intense pain, the underlying mechanisms remain unclear. Here, we show that *Tityus serrulatus* venom triggers nociceptive behaviors dependent on TRPV1, and on macrophage and neutrophil recruitment and the NFκB-mediated release of pro-hyperalgesic cytokines (TNF-α and IL-1β). This is the first study to demonstrate that TRPV1 plays a functional role in mediating localized inflammatory pain following *Tityus serrulatus* envenomation in vivo.

In our study, *Tityus serrulatus* venom evokes significant pain responses resembling grade I human envenomation (localized pain) in mice. However, more severe grade II (fever, muscle spasms, autonomic dysfunction) and grade III (respiratory distress, seizures) [7,11] responses were not observed, possibly due to interspecies differences or experimental limitations. The venom doses used were sublethal and designed to replicate grade I symptoms; thus, they may not model systemic toxicity or full immunological responses occurring in natural stings. The injected venom doses may have been insufficient to replicate higher-grade envenomation, and natural stings likely introduce additional inflammatory/immune reactions from non-aseptic conditions, potentially exacerbating and prolonging pain sensitization. Our data show that the venom produces up to 5 h of mechanical and thermal hyperalgesia. Even though we mimicked the grade I human envenomation symptoms, the behaviors did not return to the baseline.

The pro-inflammatory cytokines, TNFα and IL-1β, contribute to pain mechanisms, promote an increase in vascular permeability, and play a crucial role in cell recruitment and the activation of the innate immune response. They induce endothelial cells to express adhesion molecules, promoting leukocyte (such as neutrophils and macrophages) recruitment to sites of inflammation [37,38,39,40]. Consistent with this, *Tityus serrulatus* venom induces robust paw edema within 10–30 min after injection, persisting for up to 24 h, alongside significant recruitment of macrophages and neutrophils to the injury site [41]. Thus, TNFα and IL-1β release by *Tityus serrulatus* venom could trigger neutrophils and macrophages migration, which amplifies immune recruitment and inflammation.

To investigate the inflammatory mechanisms underlying *Tityus serrulatus* venom-induced hyperalgesia, we employed a pharmacological in vivo approach. Mice were pretreated with specific antagonists/inhibitors and subsequently evaluated using mechanical and thermal hyperalgesia tests, along with spontaneous pain behavioral assessments (licking and flinching responses). This strategy confirmed the involvement of key inflammatory mediators and transcription factor, TNF-α, IL-1β, and NFκB, respectively, in the development of *Tityus serrulatus* venom-induced hyperalgesia. These findings complement established evidence that *Tityus serrulatus* venom induces potent inflammation with rapid and sustained release of key pro-inflammatory mediators including TNF-α, IL-1β, IL-6, IL-8, IL-10, IFN-γ, GM-CSF, and nitric oxide in both animal models and human envenomation [15,16,18,19,20,21]. Our pharmacological inhibition experiments further demonstrate that blocking cytokine pathways significantly attenuates venom-induced pain, validating their role in the inflammatory pain mechanism.

Evidence supports that TNFR1 and IL-1R1 are expressed by DRG nociceptive neurons allowing neuronal sensitization by TNF-α and IL-1β. TRPV1+ neurons express TNFR1 and IL-1R1 and these receptors interfere with each other’s expression [42,43]. The activation of TRPV1 enhances TNFR1 expression while IL-1β increases TRPV1 expression by DRG neurons [42,44,45]. TNF-α nociceptor neuron sensitization involves, for instance, the phosphorylation of voltage-gated sodium channels such as Nav1.7, Nav1.8, and Nav1.9 [44,46,47], and IL-1β via IL-1R1 sensitizes nociceptor neurons by mechanisms involving protein tyrosine kinases and protein kinase C [48]. Therefore, inflammatory mediators are involved in pain induction and not solely other inflammatory events.

Our experiments demonstrate that *Tityus serrulatus* venom directly activates nociceptors, as evidenced by both immediate spontaneous pain behaviors (licking/flinching) and calcium influx in dorsal root ganglion (DRG) neurons. This nociceptive response, combined with direct DRG activation, indicates that *Tityus serrulatus* venom contains components that act through two potential mechanisms: (1) specific targeting of membrane cation channels on sensory neurons, or (2) nonspecific cytolytic action causing cation influx. These findings are supported by established evidence that *Tityus serrulatus* venom contains neurotoxins targeting specific ion channels, including Ts1, Ts2, Ts4, and Ts5 that bind voltage-gated sodium channels (Nav), and Ts6, Ts7, Ts8, Ts9, Ts15, and Ts19 fragments I/II that modulate voltage-gated potassium channels (Kv) [14,49,50,51,52,53,54]. The presence of these channel-specific toxins strongly suggests that direct interaction with neuronal ion channels contributes significantly to *Tityus serrulatus* venom’s nociceptive effects. The venom’s dual capacity to directly excite nociceptors while simultaneously triggering inflammatory cascades likely underlies the intense pain characteristic of *Tityus serrulatus* venom stings.

As we observed that *Tityus serrulatus* venom induces inflammation and pain, we questioned whether TRPV1-positive neurons, which play a key role in mechanical and thermal hyperalgesia and neurogenic inflammation [55,56,57], contribute to venom-evoked nociception behaviors. TRPV1 knockout are genetically engineered mice in which the TRPV1 gene has been disabled. *Tityus serrulatus* venom-induced mechanical hyperalgesia as well as thermal hyperalgesia and flinches behavior were significantly reduced by TRPV1 deficiency. These results are consistent with the fact that TRPV1-expressing neurons are essential for mechanical and thermal hyperalgesia and neurogenic inflammation.

Although further studies are needed to explore additional mechanisms underlying *Tityus serrulatus* venom-induced pain, our findings provide important insights into the localized pain experienced by patients. The involvement of TRPV1 channels, which enhance nociceptor activity by increasing cation influx and depolarization rate [57,58], helps explain the pain. Additionally, *Tityus serrulatus* venom-induced inflammatory pain results partly from macrophage and neutrophil recruitment, coupled with pro-inflammatory cytokine release. These dual mechanisms (direct neuronal sensitization and immune-mediated inflammation) likely contribute to the clinical challenge of managing pain in *Tityus serrulatus* stings. Our study not only elucidates key mechanisms underlying the pathophysiology of scorpion envenomation but also lays the groundwork for developing targeted therapeutic strategies that address both the nociceptive (TRPV1-mediated) and inflammatory (cytokine-driven) components of venom-induced pain. Future investigations should: (1) identify and characterize the specific *Tityus serrulatus* venom toxins responsible for neuronal and immune activation; (2) evaluate the therapeutic potential of combining TRPV1 antagonists, such as topical capsaicin analogs (e.g., Qutenza^®^), with cytokine inhibitors (e.g., etanercept or anakinra) for potential synergistic effects; and (3) validate these mechanisms using human tissue samples from envenomation cases. Importantly, clinical translation would benefit from studies correlating cytokine profiles (e.g., TNF-α and IL-1β levels) with pain severity scores in sting victims, alongside the repurposing of FDA-approved biologics for acute pain relief. Together, these approaches could bridge our mechanistic insights into practical therapeutic interventions, addressing a critical unmet need in the clinical management of scorpion envenomation.

## 4. Conclusions

Our study demonstrates, for the first time, that *Tityus serrulatus* venom induces local pain, primarily mediated by TRPV1 nociceptive neurons, macrophage and neutrophil migration, and pro-inflammatory cytokines (TNF-α and IL-1β). Therefore, targeting TRPV1, NFκB, TNF-α, and IL-1β could offer novel therapeutic strategies to alleviate scorpion sting-associated inflammation and pain.

## 5. Materials and Methods

### 5.1. Animals

Male Swiss and C57BL/6 (WT) mice (25 g, 8–12 weeks) were obtained from Londrina State University, with *Trpv1*^−/−^ mice (C57BL/6 background) provided by Ribeirão Preto Medical School [35]. Mice were housed in ventilated racks (Alesco, Monte Mor, São Paulo, Brazil) under controlled conditions (23 ± 2 °C, 12 h light/dark) with ad libitum access to sterilized food/water. After ≥48 h acclimation, mice were habituated for 1 h in experimental rooms before the experiment. Terminal procedures involved 5% isoflurane anesthesia followed by cervical dislocation. All protocols followed IASP guidelines and were approved by the Londrina State University Ethics Committee (CEUA 21366.2015.72).

### 5.2. Tityus serrulatus Venom

The *Tityus serrulatus* venom used in our study was obtained from the Butantan Institute (São Paulo, Brazil), an antivenom production center internationally recognized for its quality and standardization protocols. Briefly, *Tityus serrulatus* venom was collected from adult specimens maintained in captivity under controlled conditions (temperature 25–28 °C; relative humidity 60–80%; 12:12 h light/dark cycle). Scorpions were housed individually in transparent plastic containers with perforated lids and were provided with water and live crickets as food, following standard ethical and sanitary guidelines for arthropod maintenance. Venom was extracted by electrical stimulation of the telson (6–12 V pulses), a method previously described for safe and effective venom harvesting [59]. The collected venom droplets were immediately transferred into sterile microcentrifuge tubes kept on ice, then lyophilized, and stored at −20 °C until use.

### 5.3. Drugs and Compounds

The compounds utilized in this study included *Tityus serrulatus* venom (administered intraplantarly at doses of 0.2, 0.6, and 2.4 μg in 20 μL), Etanercept (10 mg/kg in 200 μL sa-line, given 48 h and again 1 h before venom exposure; CAS 185243-69-0; Wyeth Indústria Farmacêutica Ltda, Itapevi, São Paulo, Brazil), Interleukin-1 receptor antagonist (IL-1ra; 30 mg/kg in 200 μL saline, administered 30 min prior to stimulus; CAS 566914-00-9; National Institute of Biological Standards and Control, Potters Bar, Hertfordshire, UK), and Pyrrolidine dithiocarbamate (PDTC; 100 mg/kg in 100 μL of 2% DMSO in saline, given 30 min before the stimulus; CAS 5108-96-3; Santa Cruz Biotechnology, Dallas, Texas, United States), as described by Ferraz et al. (2015) [38]. *Tityus serrulatus* venom, etanercept, and IL-1ra were prepared in sterile saline, while PDTC was dissolved in a 2% DMSO-saline mixture. The dosages were based on previously validated protocols established in our laboratory and in the literature [38,39,60,61,62].

### 5.4. Experimental Protocols

Mice were given intraplantar (i.pl.) injections of *Tityus serrulatus* venom (0.2, 0.6, or 2.4 μg/20 μL per paw) or vehicle control. Mechanical and thermal hyperalgesia were assessed at 0.5, 1, 3, and 5 h after injection, while spontaneous pain behaviors were observed for 30 min following administration of the highest venom dose (2.4 μg). Pharmacological treatments, including vehicles, etanercept, IL-1 receptor antagonist (IL-1ra), and pyrrolidine dithiocarbamate (PDTC), were administered intraperitoneally (i.p.) or subcutaneously (s.c.). Cytokine concentrations were measured at 0.5, 1, and 3 h after injection. Myeloperoxidase (MPO) and N-acetyl-β-D-glucosaminidase (NAG) enzyme activities, indicative of neutrophil/macrophage and macrophage presence, respectively, along with histological analyses, were performed at 5 h after injection. Immunofluorescence staining of DRG neurons was conducted at 3 h. In ex vivo experiments, DRG neurons isolated from naïve mice were cultured overnight and then stimulated with capsaicin or venom to assess calcium influx through imaging.

### 5.5. Mechanical Hyperalgesia Test

Mechanical hyperalgesia was evaluated using an electronic von Frey aesthesiometer (Insight, Ribeirão Preto, SP, Brazil), following previously established procedures [63]. Prior to testing, mice were habituated for at least one hour in individual acrylic cages (12 × 10 × 17 cm) with wire grid floors in a quiet environment. Stimuli were delivered only when the animal remained still (i.e., no movement, defecation, or resting of the paw). A 0.5 mm^2^ polypropylene-tipped probe was applied perpendicularly to the center of the right hind paw with increasing pressure until a withdrawal accompanied by flinching was observed. The withdrawal force (in grams) was recorded automatically, based on three measurements per time point, by a single blinded experimenter to maintain consistency. Results are presented as the change in withdrawal threshold (Δ threshold = post-injection average − baseline average) measured at 0.5, 1, 3, and 5 h following venom administration.

### 5.6. Thermal Hyperalgesia Test

Thermal hyperalgesia was assessed using a hot plate apparatus (IITC Life Science, Woodland Hills, CA, USA) set at 52 ± 1 °C, following standard protocols [39,64,65]. Mice were placed inside a transparent acrylic chamber on the heated surface, and the latency to respond, indicated by hind paw licking or flinching, was recorded in seconds. To prevent injury, a cutoff time of 15 s was enforced. Measurements were taken both before and after venom administration by an experimenter blinded to the treatment groups. Results are reported as the thermal response threshold in seconds.

### 5.7. Spontaneous Pain Behavior Test

Spontaneous pain behaviors were evaluated by measuring the number of paw flinches and the total duration of licking within 30 min after intraplantar injection of *Tityus serrulatus* venom into the right hind paw. Immediately following injection, each mouse was placed individually into an observation chamber, where nociceptive responses were recorded, specifically: (1) the total count of paw flinches and (2) the cumulative licking time (in seconds) of the affected paw, using established protocols [39,66].

### 5.8. Myeloperoxidase (MPO) and N-Acetylglucosaminidase Activity (NAG) Assays

Myeloperoxidase (MPO), a marker for neutrophils and macrophages, and N-acetyl-β-D-glucosaminidase (NAG), a macrophage-specific marker, activities were assessed as previously described [39]. Paw skin samples were collected 5 h after venom injection and homogenized in ice-cold 50 mM potassium phosphate buffer (pH 6.0) containing 0.5% hexadecyltrimethylammonium bromide (HTAB) using a Tissue-Tearor (Biospec^®^, Bartlesville, OK, USA), then centrifuged at 16,100× *g* for 2 min. For MPO measurement, 15 μL of the supernatant was combined with 200 μL of potassium phosphate buffer containing o-dianisidine dihydrochloride (0.167 mg/mL) and hydrogen peroxide (0.015%), and absorbance was read at 450 nm using a Multiskan GO spectrophotometer (Thermo Fisher, Waltham, MA, USA). NAG activity was determined by incubating 10 μL of supernatant with 4-nitrophenyl N-acetyl-β-D-glucosaminide (2.24 mM) in phosphate buffer at 37 °C for 10 min; the reaction was terminated with glycine buffer (pH 10.6), and absorbance was measured at 400 nm. Results are expressed as the number of neutrophils (MPO) or macrophages (NAG) per mg of tissue [39].

### 5.9. Cytokine Measurement

Paw skin samples were collected at 0.5, 1, and 3 h after venom or control injection and homogenized in 500 μL of buffer supplemented with protease inhibitors. The resulting supernatants were analyzed for TNF-α, IL-1β, and IL-33 concentrations using commercial ELISA kits (eBioscience^®^, San Diego, CA, USA), following the manufacturer’s instructions [39]. In brief, 96-well plates were coated overnight at 4 °C with antibodies specific to each cytokine, then blocked and incubated with standards or samples overnight. Afterward, biotinylated detection antibodies were added for 1 h at room temperature, followed by a 30-min incubation with avidin-HRP. The enzymatic reaction was developed using o-phenylenediamine for 30 min, stopped with sulfuric acid, and absorbance was measured at 450 nm. Cytokine levels were expressed as picograms per 100 mg of tissue [39].

### 5.10. Paw Tissue Histology

Paw tissues were fixed in 10% formaldehyde, embedded in paraffin, and sliced into 7 μm longitudinal sections. These sections were stained with hematoxylin and eosin (H&E) for morphological examination [39,67]. Four sections per mouse were captured using a light microscope at 40× magnification (Olympus CX31RTSF, Tokyo, Japan), with the same light intensity for all groups. Image analysis was conducted with ImageJ 1.44 software, utilizing threshold processing on RGB images [Hue 55:190, saturation 78:255 and brightness 0:255 and threshold of 6.59% (0:50)]. All images were analyzed in their original, uncropped form.

### 5.11. Immunofluorescence

Dorsal root ganglion (DRG) neurons were collected 3 h after *Tityus serrulatus* venom injection, following intracardiac perfusion with PBS and 4% paraformaldehyde (PFA). The tissues were post-fixed overnight in 4% PFA at 4 °C, then cryoprotected in 30% sucrose for 3 days at 4 °C before being embedded in OCT compound for cryosectioning into 10 μm sections. Sections were blocked with 5% BSA and 0.3% PBST, then incubated overnight at 4 °C with primary antibody against phosphorylated NFκB p65 (mouse anti-pNF-κB p65, #sc-136548, Santa Cruz Biotechnology, Dallas, TX, USA; 1:200). After washing with PBST, sections were incubated at room temperature for 50 min with Alexa Fluor 647-conjugated goat anti-mouse secondary antibody (1:500). Nuclear staining was performed with DAPI (1:500). Following final washes, slides were mounted in Fluoromount-G. Imaging was carried out using a Leica TCS SP8 confocal microscope with a 20× objective, 1.0 zoom, 405 nm (intensity: 4%, gain: 729.3), HyD (645–695 nm) gain: 60, scan mode xyz, scan speed 100 Hz. All images were processed and presented in maximum projection without background removal. Images were analyzed using Leica EL6000 software. The results are presented as the percentage of double-positive cells based on manual counting [32].

### 5.12. Calcium Imaging

DRG neurons from naïve mice were isolated by enzymatic digestion using collagenase A (1 mg/mL) and dispase II (2.4 U/mL) in HEPES-buffered saline for 15 min at 37 °C. The cell suspension was centrifuged at 300× *g* for 5 min and resuspended in DMEM supplemented with 10% FBS and DNase I. Following mechanical dissociation and BSA gradient centrifugation at 300× *g* for 10 min, cells were plated at a density of 5000 neurons per dish in Neurobasal-A medium containing 50 ng/mL nerve growth factor (NGF) on laminin-coated plates and incubated overnight. For imaging, neurons were loaded with 1.2 μM Fluo-4 AM (30 min, 37 °C), washed with HBSS, and imaged on a Leica SP8 confocal microscope using identical gain and exposure settings across all treatment groups to ensure comparability. Calcium recordings consisted of a 2-min baseline, 2-min stimulation with either capsaicin (1 μM) or *Tityus serrulatus* venom (2.4 μg/mL), followed by 2-min depolarization with 40 mM KCl. Recording was carried out using a Leica TCS SP8 confocal microscope with a 20× objective, 1.0 zoom, and 488 nm (intensity: 3%, gain: 750, scan mode: xyt, scan speed: 400 Hz). Fluorescence intensity was quantified using LAS X software 3.7.6.25997, and raw fluorescence data were analyzed without background subtraction [33,68].

### 5.13. Statistical Analysis

All the results are expressed as mean ± SEM (6 animals per group), except for: (1) the systemic treatment of NFκB, TNF-α, and IL-1β inhibitors in mechanical and thermal hyperalgesia tests, where we used six animals in total, with four selected for spontaneous pain behavior test (flinch and licking experiments); and (2) immunofluorescence assay, which used four animals per group. The minimum sample size needed to have power to detect an effect was analyzed by G*power version 3.1.9.4. We performed two independent experiments. Prior to statistical analysis, datasets were evaluated for normality using the Shapiro–Wilk normality test and Brown–Forsythe homogeneity of variance. When data met the assumptions of normality and homogeneity, parametric tests were applied. For experiments involving multiple time points and comparisons across three or more groups (e.g., mechanical and thermal hyperalgesia), a two-way repeated measures ANOVA followed by Tukey’s post hoc test was used. In cases where only one point was assessed and more than two groups were compared, one-way ANOVA with Tukey’s post hoc test was performed, provided the data were normally distributed and homogeneous. For comparisons between two groups at a single time point, the Student’s t-test was used under the same statistical assumptions. Significance was determined at *p* < 0.05. All analyses were conducted using GraphPad Prism 10.

## Data Availability

The original contributions presented in this study are included in the article. Further inquiries can be directed to the corresponding author.
